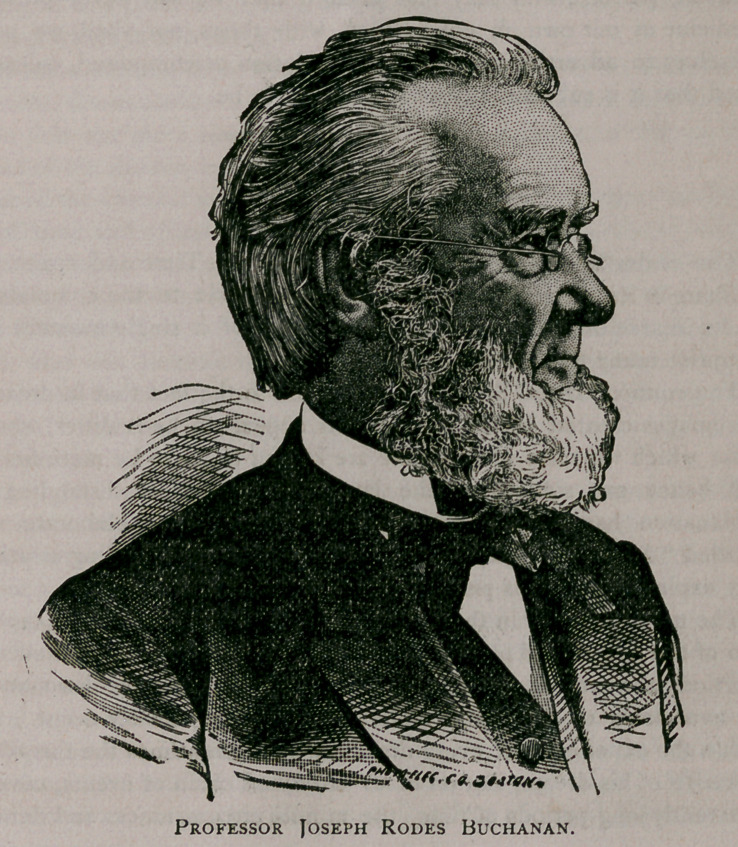# Prof. Joseph Rodes Buchanan

**Published:** 1886-11

**Authors:** 


### HALL'S JOURNAL OF HEALTH

TRUTH DEMANDS NO SACRIFICE. ERROR CAN MAKE NONE.

VOL. 33. NOVEMBER, 1886. No. 11.

PROFESSOR JOSEPH RODES BUCHANAN.

### PROF. JOSEPH RODES BUCHANAN.

It is unfortunate for the living, that the labors of those whose inventions and discoveries outstrip the progress of the age and carry the world into new fields of knowledge, are scarcely ever appreciated as they deserve to be, until the head that planned and the hand that executed them, are no longer available. Then, indeed, the recognition comes too late to assist their efforts or sustain the wasted energies of a life wearied by unceasing endeavor and disappointment.

From time out of mind it has been the fate of original genius to be the subject of neglect, if not of persecution or ridicule, on the part of those who should be foremost to recognize and encourage its real worth.

Oftentimes far in advance of accepted beliefs and the dull levels of religious dogma, it has been made to feel its iron hand in periods when the dominating power discouraged general education and prescribed bounds to philosophical research, beyond which no investigator could safely venture. It was so when Socrates gave up his life at the demand of the Athenean rulers, and so was it when Galileo was thrown into prison with a like fate impending over him. These are only representative instances of many others equally cruel and unreasonable. Faust, the inventor of printing from engraved wooden blocks or forms who, by this simple means was able to multiply with a celerity quite incomprehensible to the common mind, and at greatly reduced rates, copies of the "holy bible;" was forced to abandon his occupation and flee his country to save his life, under the imputation of being in league with the "evil one" in this novel enterprise. So at an earlier period, Roger Bacon who, in the seclusion of his monastry devoted himself to experimental philosophy, and invention, was accused by his brother religionists, upon like charges, and thrust into a prison, whose narrow walls confined for ten years, the most profound thinker of his period, for no other cause than his unappreciated discoveries, for which, even the present age is greatly indebted.

So was it with Peter of Apono, a renowned philosopher, mathematician, astrologer and physician of the same period. Upon being accused of magic by his ignorant brethren and having eluded their vengeance by flight, only to die an untimely death, his bones were ordered to be exhumed and burned as a measure of *retributive justice*, but as this could not be carried out, his presecutors contented themselves with burning his effigy. Few indeed are they who, having made discovery of some of the more wonderful and hitherto unknown laws of nature, of sufficient importance to elicit public attention, have been so fortunate as to overcome the opposition of the learned, or the distrust and hostility of the ignorant. Newton, Harvey, Mesmer, Gall, Spurzheim, Franklin and Fulton did not escape this *devil's toll*, at the gateway of their splendid achievements.

It affords us pleasure to be able to note as an evidence of the growing liberality of the age, a remarkable exception to this too prevalent hostility, in the instance of one of our own citizens whose psycological and physiological discoveries during more than a half century of unremitting labor despite the opposition of a stagnant conservatism, have drawn to him the generous recognition and commendations of some of the ablest scientists of this and other countries. We allude to Doctor Joseph Rodes Buchanan, now residing in the City of Boston, a very accurate likeness of whom will be found in the present number of the JOURNAL.

Doctor Buchanan, who is now in his seventy-second year, is a native of Kentucky. Many years ago he became widely known as the editor and publisher of "Buchanan's Journal of Man," a serial of marked ability, originality and scholarship, in the direction in which the extraordinary powers of his mind, have been chiefly directed—the science of man, and the best methods of human progress. We say "extraordinary" for the most remarkable quality of his mind, is its originality and independence. He has never given his patient attention to any subject without enlarging its boundaries beyond the limits of his predecessors.

Even upon those great political questions, which agitate society to its foundations, Dr. Buchanan has always been an original thinker, having studied political economy at a very early age and watched with intense interest the experiments of Robert Owen in America, in 1825-26.

The doctrine of the right of the people to the land on which the nation lives, originated with him, and was promulgated at Cincinnati in 1848 in an able essay of thirty pages in "Hine's Herald of Truth," in which he traced the logical consequence of the doctrine, and urged the resumption by the people of their eminent domain, to be sustained by a small and progressively increasing tax upon land, thus making the nation virtually the landlord, capable under such a system of doing an unlimited work in philanthropy. He has lived to see with great pleasure the adoption of similar doctrines by well known statesmen, making them the leading questions of the age.

Returning to his native state, when the whole country was convulsed by internal strife, Dr. Buchanan was at once forced into leadership, and as Chairman of the Democratic State Committee achieved great prominence for his statesmanlike policy of uniting all orders of men in a single effort of national *solidarité* and peace, and having assisted in its accomplishment, he declined all further political honors in order to resume those scientific investigations, with which his name is inseparably connected.

Before attaining his majority, Dr. Buchnnan entered Transylvania University as a student of medicine, and at the very threshold of his professional career, became profoundly interested in the structure and functions of the brain. Having made himself familiar with the works of Gall, he was led not alone by research and experiment, but as well by intuition, to accept their teachings, and pushing on with absorbing interest from the utmost point attained by that emnient investigator, he was permitted to explore the innermost recesses of the human battery, and thus to achieve for phrenology as an element of anthropology, what Galileo achieved for astronomy. He has done even more, for pressing on beyond the limits of any of his predecessors, he has made original discoveries of unspeakable importance, in a field hitherto unoccupied, founding new sciences, and giving them distinguishing names indicative of their characteristics. PSYCHOMETRY and SARCOGNOMY, are the offspring of his laborious investigations guided by his genius. The former as its name implies is emphatically a science of the soul—the name being formed from Greek words which signify soul measuring. It is fully illustrated and demonstrated as a science in the "MANUAL OF PSYCOMETRY" published by Dr. B. in 1885 showing that the powers of the human soul have never yet been understood by the learned. and that by their proper development we shall have an era of progress in science and philosophy so entirely different from the past as to be justly termed "the dawn of a new civilzation."

SARCOGNOMY shows the sympathy of the body with the brain and soul, and consequently explains both its psychic significance and the laws of health and disease in their joint operation upon soul, brain and body. This feature of Sarcognomy is explained in the " THERAPEUTIC SARCOGNOMY " published by Dr. B. in 1884, but as the whole edition was sold in a few months, copies of this work cannot now be obtained. Therapeutic Sarcognomy gives the exact scientific method of treating the human body by electricity and by animal magnetism or massage ; and courses of instruction in this, are given every year by Dr. Buchanan in Boston. Such a course is now in progress. The artistic applications of Sarcognomy to statuary and painting have not been published. These sciences which revolutionize Physiology and Psychology are foreign to the mental atmosphere of our Universities and have had no collegiate presentation except by Dr. B. himself in the four medical colleges in which, between 1845 and 1886 he has occcupied the professorship of Physiology and Institutes of Medicine, yet wherever he has presented them, they have received thc endorsement of his listeners.

Among the theologians and literati of Boston, forty years ago, the Rev. John Pierpoint, the well remembered scholar and poet, spoke of Dr. Buchanan discoveries as the most wonderful of the age, and in the course of his poem on progress delivered at the 150th, anniversary of Yale College, comparing Psychometry and photography, he said :

But much DAGUERRE as has thy genius done
In educating thus Latona's Son,
In thus educing in the god of light
The power to paint so at a Single Sight,
BUCHANAN has transcended thee as far
As the sun's face outshines the polar star.

The very page that I am tracing now
With tardy fingers and a careworn brow,
To other brows by other fingers press't,
Shall tell the world, not what I had been deem'd,
Nor what I passed for, nor what I had seem'd,
But what I *was* ! Believe it friends or not,
To this high point of *progress* have we got,
We stamp ourselves on every page we write !
Send you a note to China or the pole—
Where'er the wind blows, or the waters roll—
That note conveys the measure of your soul ."

It is the doctrine of Psychometry demonstrated by many thousand experiments, that those who possess the psychometric faculty, are able by touching any manuscript to feel and describe the character of the writer. The late Prof. Denton, who fell a victim to his devotion to national science; in his most remarkable work, " The Soul of Things," characterises Doctor Buchanan "as one of the most vigorous thinkers, boldest writers, and greatest discoverers of this or any age." However extravagant these words may appear to such as are unacquainted with Doctor Buchanan and his works, those who know him most intimately, will readily concede them to be a measure of praise by no means overstrained. From early boyhood to the present hour Professor Buchanan's life has been an exceedingly laborious one. He has repeatedly drawn public attention to his wonderful discoveries, which, for the most part have been so original and startling, as almost to obscure the master spirit that wrought and fashioned them to the comprehension of man.

Aside from his published works in which are included " Buchanan's Journal of Man," his " System of Anthropology," " The New Education," and his " Manual of Psychometry," the Doctor has in store a large body of manuscript upon kindred subjects, to which he has almost exclusively devoted himself for the past ten years, and which he is now preparing for publication. In respect to these labors we cannot do better than to quote from Professor Britton's Biography of Doctor Buchanan as incorporated into the published record of Louisviile's distinguished men. Speaking of the generous recognition of Dr. Buchanan's discoveries by Mr. Pierpoint and by scientific committees, he adds:

" Such has always been the language of profound thinkers who have become acquainted in detail with Dr. Buchanan's system of anthropology." The faculty of the Indiana State University (Dr. Wylie, president), Prof. Caldwell, the virtual founder of the old University Medical School of Louisville, and others equally well versed in physiological lore, have acknowledged the great value of Dr. B's discoveries, in terms of unstinted praise.

After five years spent in the investigation and propagation of the new anthropology, Dr. Buchanan accepted the professor's chair of " Physiology and the Institutes of Medicine " in the Electric Medical Institute of Cincinnati, which he occupied for ten years, during a considerable portion of which time he was dean of the faculty. His peculier discoveries and new views of physiology constituted an attractive feature of the school, which rapidly grew to a success surpassing its older rivals in that city.

In addition to the duties of his professorship Dr. Buchanan edited a medical magazine and published for five years "Buchanan's Journal of Man," chiefly of original matter. He also published an edition of two thousand copies of his "System of Anthropology," which was but a synopsis of four hundred pages. The complete system unfolds the laws of mind and explains its operations through the brain and body, the full developement of which will require at least ten volumes, in the preparation of which Dr. Buchanan is at present diligently engaged as stated by Dr. Brittan. "Their scope embraces a review of all the great systems and fragments of philosophy of the present and past centuries; a precise view of "Mental Philosophy," embracing not only the functions of the brain, but the categorical or *a priori* demonstration of the faculties; a complete system of "Cerebral Physiology," supplying the great *hiatus* in systems of physiology (which almost ignore the brain) and laying the foundations of a complete philosophy of therapeutics; a system of "Sarcognomy," explaining the development of the body and its relations to the soul; a system of "Pathognomy," giving the laws of expression and oratory, with the mathematical basis of all relations between mind and matter; a system of "Physiogomy," not based on empirical observation, but on laws of mathematical certainty. All the fundamental laws of the fine arts and æsthetics are comprised in the systems of "Pathognomy" and "Sarcognomy." A volume will be devoted to "Psychometry," another to "Insanity," and another to the marvelous facts of "Psychology," subjects which from their vast extent, have never been fully developed in his lectures.

In these works Dr. Buchanan has shown their application to human improvement by "The New Education," published in 1882 and now in its third edition. This woık is about to be republished in the Japanese language at Tokio. The Rev. B. F. Barrett himself an eminent author says of it. "It contains more and higher wisdom on the subject of which it treats, than all the other books ever written on education." "His great work will live (said Dr. Strickland) when all bigotted opposers are forgotten." Dr. B. has been honored abroad, as well as at home. His "Therapeutic Sarcognomy," has been translated into Spanish, and his "Manual of Psychometry," is being translated into French at Paris.

We speak advisedly when we say, that the one great aim of Doctor Buchanan's ripe old age, is to leave to the world in enduring form, the results of his manifold labors. But he desires also to reach the liberal minds among his contemparies and to apply his grand discoveries to social progress. To do this he proposes early in 1887 to renew the publication of "Buchanan's Journal of Man," an announcement which will be hailed with pleasure by thousands.

Our limited space will not permit us to enter further into the detail of Doctor Buchanan's extraordinary career, however enticing the subject and profitable to our readers. This has already been done in the biographical sketches by Prof. Brittan and the Rev. W. P. Strickland D.D., but the complete work remains for the historian, when the name of Joseph Rodes Buchanan shall be inscribed with commendable pride, upon the glittering roll of our nation's honored sons.

## Figures and Tables

**Figure f1:**